# α-Defensins Promote *Bacteroides* Colonization on Mucosal Reservoir to Prevent Antibiotic-Induced Dysbiosis

**DOI:** 10.3389/fimmu.2020.02065

**Published:** 2020-09-09

**Authors:** Jiayao Ou, Shaonan Liang, Xue-Kun Guo, Xiaoyu Hu

**Affiliations:** ^1^Institute for Immunology and School of Medicine, Tsinghua University, Beijing, China; ^2^Tsinghua-Peking Center for Life Sciences, Tsinghua University, Beijing, China; ^3^Beijing Key Laboratory for Immunological Research on Chronic Diseases, Tsinghua University, Beijing, China

**Keywords:** antimicrobial proteins, α-defensins, microbiota colonization, mucosal reservoir, intestinal epithelial cell

## Abstract

In addition to their established functions in host defense, accumulating evidence has suggested an emerging role for antimicrobial proteins (AMPs) in shaping commensal microbiota. However, the role of α-defensins, the most abundant AMPs of intestine, in regulating microbial ecology remains inconclusive. Here, we report that α-defensins promote commensal *Bacteroides* colonization by enhancing bacterial adhesion to the mucosal reservoir. Experiments utilizing mice deficient in matrix metalloproteinase 7 (MMP7), the α-defensin–activating enzyme, with rigorous littermate controls showed that α-defensin deficiency did not significantly influence steady-state intestinal microbiota. In contrast, α-defensins are essential for replenishment of commensal *Bacteroides* from the mucosal reservoir following antibiotics-induced dysbiosis, shown by markedly compromised recovery of *Bacteroides* in *Mmp7*^−/−^ animals. Mechanistically, α-defensins promote *Bacteroides* colonization on epithelial surfaces *in vivo* and adhesion to epithelial cells *in vitro*. Moreover, α-defensins unexpectedly does not show any microbicidal activities against *Bacteroides*. Together, we propose that α-defensins promote commensal bacterial colonization and recovery to maintain microbial diversity upon environmental challenges.

## Introduction

The mammalian gastrointestinal tract is under continuous exposure to trillions of microorganisms (microbiota) that play fundamental roles in maintenance of gut homeostasis, modulation of the immune system, facilitation of digestion, and regulation of distant organ functions in physiology and disease (1, 2). Although day-to-day variabilities in diet and exposures to diverse environmental factors could influence microbiota ecology, community compositions of the adult gut are relatively stable because of their ability to recover from the reservoirs of bacterial cells (3, 4). For instance, severe perturbations of the gut microbiota by antibiotics lead to a low-diversity consortium, yet after a period of recovery, membership, and relative abundance largely resemble the pretreatment state (5). Certain species that have been diminished to undetectable levels in stool by antibiotic treatment are later recovered (3, 5, 6), supporting the notion that there exist reservoirs that protect bacterial cells and reseed them to the intestinal lumen (3). Intestinal crypts, mucus layers, and the appendix have been proposed to act as mucosal reservoirs to sustain community diversity (3). For example, human commensal species *Bacteroides fragilis* colonizes to the deep colonic crypts for long-term resilience to intestinal perturbations such as antibiotic treatments (7). The mucus layers not only serve as nutrients for bacteria but also provide attachment sites that are protected from the fecal streams (8).

In addition to the bacterial reservoirs, endogenous components of the mucosal system are also essential players in shaping microbiota ecology. For example, antimicrobial proteins (AMPs) secreted by intestinal epithelia cells help maintain proper segregation of microbiota from the epithelial surfaces (9). Intriguingly, besides their protective functions, several recent studies imply that AMPs could also be detrimental to host defense by promoting colonization of certain enteric pathogens (10, 11). Such emerging evidence begins to expand the conventionally defined microbicidal functions of AMPs and unveil the multifaceted nature of interactions between AMPs and microbes.

α-Defensins are the most abundant and diverse AMP families in the gut (12), expressed as inactive propeptides and subsequently processed to the bioactive proteins by MMP7 in mice and by trypsin in humans (13, 14). The active α-defensins contribute to innate host defense against enteric pathogens in the gut (15). Moreover, dysregulation of α-defensins has been observed under pathogenic conditions such as inflammatory bowel disease (IBD) (16–18). For example, patients with Crohn disease of the ileum harbor reduced levels of α-defensins in their intestinal mucosal extracts (18), which is associated with inflammation in ileal Crohn disease (17). It is noteworthy that α-defensins have been implicated in regulating steady-state commensal bacterial compositions in a study using *Mmp7*^−/−^ and *DEFA5*-transgenic mice (19) yet there exist scarce follow-up studies to either verify or challenge such conclusions. More importantly, the mechanisms underlying the interaction between α-defensins and commensal bacterial community remain unclear. Our previous study has suggested a crucial role for α-defensins in protecting host against infections by enteric bacterial pathogens upon nutrient deprivation, whereas this phenotype was not revealed in nutrient sufficient conditions (20), implying that functions of α-defensins may be tightly coupled to environmental changes. Therefore, to investigate the potential influences of α-defensins on microbiota, we performed gut microbiota phylogenetic analyses using rigorous littermate controls under homeostatic conditions or upon environmental challenges. Data from microbial analyses and functional studies demonstrated lack of significant differences in the fecal or terminal ileum microbiota of *Mmp7*^+/+^ and *Mmp7*^−/−^ littermates at homeostasis, yet revealed a previously unappreciated role of α-defensins in facilitating microbiota recovery from antibiotics-induced dysbiosis by promoting bacterial colonization on the mucosal reservoirs such as epithelial surfaces.

## Methods

### Mice

The wild-type C57BL/6J and *Mmp7*^−/−^ mice were purchased from the Jackson Laboratory, USA, and maintained under specific pathogen-free conditions with a 12-h light–dark schedule. The *Mmp7*^+/+^ and *Mmp7*^−/−^ littermates were generated by crossing *Mmp7*^+/−^ and *Mmp7*^+/−^ mice on the C57BL/6J background. The littermates were cohoused after weaning and then separated until experiment. All the experimental mice were 6- to 8-week-old. Animal studies were conducted under protocols approved by the Institutional Animal Care and Use Committee at Tsinghua University.

### Quantitative PCR Analysis of Commensal Bacteria and Pathogenic Bacteria

The *Mmp7*^+/+^ and *Mmp7*^−/−^ mice were pretreated with vancomycin (5 mg/25 g mouse/day; Meilunbio, China) by oral gavage for 2 days. The small intestine and colon feces were collected for microbiota analysis at the indicated time points. Bacterial DNA extraction and analysis were carried out as previously described (20). Briefly, the fresh feces of enteric cavity in small intestine and colon were collected, and microbial DNA was extracted with the Stool Genomic DNA Kit (CoWin Biosciences, China). The abundances of specific intestinal bacterial groups were measured by quantitative polymerase chain reaction (qPCR) with FastSYBR mixture (CWBIO, China) and specific 16S rDNA primers (Table S1).

### *B. fragilis* Colonization of SPF Mice

*B. fragilis* (ATCC, USA) was expanded in liquid brain–heart infusion (BHI; BD Bioscience, USA) medium at 37°C under anaerobic condition. The mice were treated with metronidazole (100 mg/kg; Solarbio, China) by oral gavage every day and ciprofloxacin dissolved in drinking water (0.625 mg/mL; Solarbio, China) for 7 days. Two days after the cessation of antibiotics treatment, *Mmp7*^+/+^ and *Mmp7*^−/−^mice were orally administrated with a single inoculum of 1 × 10^8^ colony-forming units (CFUs) of *B. fragilis*. One day after gavage, the mice were sacrificed for determination of bacteria.

### 16S rDNA Sequencing and Analysis

The 16S rDNA sequencing and analysis were performed as previously described (21). Briefly, the bacterial DNA was extracted as described above. The distinct regions (16S V4) were amplified by specific barcoded primers with Phusion® High-Fidelity PCR Master Mix (New England Biolabs) and purified with Qiagen Gel Extraction Kit (Qiagen, Germany). The TruSeq® DNA PCR-Free Sample Preparation Kit (Illumina, USA) was used to generate sequencing libraries, and the quality was assessed with the Qubit@ 2.0 Fluorometer (Thermo Fisher Scientific, USA) and Agilent Bioanalyzer 2100 system. The libraries were sequenced on an Illumina HiSeq2500 platform, and 250-bp paired-end reads were generated. The raw tag sequences from this study were processed and quality filtered using the default parameters of QIIME version 1.7.0 (22). The tags were analyzed by Gold database, and the chimera sequences were detect by UCHIME algorithm (23). More than 97% similarity of sequences were assigned as the same operational taxonomic units, and these sequences were classified and annotated by GreenGene Database and RDP classifier (24, 25). Full DNA-seq data have been deposited in NCBI's BioProject and are accessible through the accession number PRJNA627093.

### Histology and Immunohistochemistry

The small intestine and colon tissue were fixed with 4% paraformaldehyde. The tissues were embedded in paraffin and cut in 5-μm sections. Tissue sections were stained with hematoxylin and eosin. For the immunohistochemistry staining, the tissues were incubated with anti–*B. fragilis* antibodies (CUSABIO, China). The slides were then washed with 0.1% TBS-Tween for three times before incubation with secondary antibodies, which were conjugated with Alexa Fluor 647 (1:50; Abcam, UK) for 2 h at 4°C. Stained slides were washed again in TBS before costaining with DAPI (Beyotime Biotechnology, China) and mounting with Fluoroshield Mounting Medium (Abcam, UK). For analysis of occupation of mucosal niches by *B. fragilis*, the quantification was defined as the number of bacterial cells per 0.01 mm^2^ (0.1 × 0.1 mm) area from the surface of small intestine and colon epithelia cells to lumen. Four random areas were counted per histological sections from four to six mice of each group.

### Acid/Urea-Polyacrylamide Gel Electrophoresis

Detection of mature α-defensins by acid/urea–polyacrylamide gel electrophoresis (AU-PAGE) was performed essentially as described previously (20, 26). In brief, the tissues of small intestine were longitudinally opened and washed with cold phosphate-buffered saline (PBS). The tissues were divided into 1-cm segments and shaken in 5 mM EDTA with PBS at 4°C for 70 min. The fragments were discarded, and the solution was filtered with a strainer (70 μm) to enrich for crypts. The crypts were divided into two parts at the ratio of 1:4. The 1/5 part was lysed by RIPA buffer (Beyotime Biotechnology), and total protein content was determined by BCA Protein Assay (Pierce Biotechnology, USA). The other part was lysed with AU-PAGE loading solution (33% acetic acid, 5% 2-mercaptoethanol, 9 M urea). Equal amounts of proteins were electrophoresed on a 12.5% AU-PAGE gel followed by immunoblotting analysis using rabbit polyclonal serum (1:5,000) against mouse mature α-defensin 5 peptide.

### *In vitro* Adhesion Assay and Scanning Electron Microscopy

The bacteria adhesion assay was performed essentially as described previously (11). Briefly, 1 day before assays, 10^5^ HeLa cells (ATCC) *or CMT-93 cells (ATCC* CCL-223) seeded into 24-well plates. One hour before inoculation with bacteria, the medium was changed into serum-free medium. The *B. fragilis* (1 × 10^5^ CFUs) was incubated with mouse mature α-defensin 5 (10 μM, Purity≥ 95%; Mimotopes Pty Ltd, China) for 15 min and then the bacteria were added to the cells together with mature α-defensin 5. Bacteria were centrifuged (2,000 rpm, 10 min, RT) onto HeLa cells *or CMT*-93 cellsto synchronize the inoculation. Twenty minutes later, the plates were washed with PBS for 3 times. For adhesion assay, the cells were lysed with 0.1% Triton/H_2_O, and the quantification of *B. fragilis* was analyzed by qPCR. For scanning electron microscopy (SEM) analysis; the cells and bacteria were fixed by 2.5% glutaraldehyde at 4°C overnight. The fixed specimens were dehydrated in graded ethanol, treated with tertiary butanol for 10 min twice and freeze-dried. The specimens were then coated with gold–palladium beads and were photographed using an FEI Quanta 200 scanning electron microscope at 15 kV.

### Isolation of Mouse Intestinal *Bacteroides*

The wild-type mice were pretreated with streptomycin (0.5 mg/mL; Tokyo Chemical Industry, Japan) for 5 days. The colonic feces were collected and dissolved with 1 mL PBS. PBS-diluted feces were mixed well and centrifuged at 400 g for 5 min to remove larger particles from bacteria, and then the supernatant was diluted into different concentration and plated to the Wilkins–Chalgren anaerobic agar (OXOID, USA), which contained 50 μg/mL different antibiotics [kanamycin (Solarbio, China), neomycin (Amresco, USA) or streptomycin] and cultured in anaerobic box at 37°C for 3 days. The purity of bacterial colonies was determined by qPCR analysis of 16S rDNA. Then the single colonies were streaked at least three times onto fresh agar plates with antibiotics. Culture purity was ensured by observing colony morphology. For identification and phylogenetic analysis of isolates, DNA was extracted from pure cultures, and 16S rRNA genes were amplified and sequenced. These sequences were classified and annotated by GreenGene Database (24).

### Antimicrobial Activity Assays *in vitro*

The mouse mature α-defensin 5 was dissolved in PBS (4.5 μM) and incubated with 500 to 1,000 CFUs of bacteria [*Listeria monocytogenes–OVA* (a gift from Chen Dong at Tsinghua University), *S. typhimurium* (NCTC, UK), or *B. fragilis*] for 1 h at 37°C. The solutions were plated on SS agar (BD Bioscience, USA), LB, or BHI plates. For the single clone of S24-7 family of Bacteroidetes, 1 × 10^4^ CFUs of bacteria were incubated with 4.5 μM mouse mature α-defensin 5 and plated on Wilkins–Chalgren anaerobic agar in anaerobic box. Bacterial viability was determined by CFU counting and normalized against the viability observed with mock (PBS) treatment. The colon feces of WT mice were collected, and 10 mg feces were suspended with 1 mL PBS. One microliter of suspension was incubated with the same concentration of mature α-defensin 5 as above. And the mix bacteria were plated on Wilkins–Chalgren anaerobic agar in anaerobic box and analyzed by qPCR.

### Analysis of Mature α-Defensin 5–Mediated Killing of Pathogens After Preincubation With *B. fragilis*

Mouse mature α-defensin 5 (4.5 μM) was preincubated with 1 × 10^6^ CFUs of *B. fragilis* in 200 μL PBS in anaerobic conditions at 37°C for 1 h. The mixture of preincubated α-defensin 5 was incubated with ~500 CFUs of bacteria (*L. monocytogenes–OVA, S. typhimurium*, and *Citrobacter rodentium*) in 200 μL PBS at 37°C for 1 h. The PBS or *B. fragilis* alone was used as controls. Then, the solutions were plated on BHI agar, chromogenic agar, or MacConkey agar (OXOID, UK) plates, respectively. The survived CFUs of bacteria were counted after cultivation.

### Analysis of the Integrity of Mouse Mature α-Defensin 5 *in vitro*

Bacteria 1 × 10^6^ CFUs [*S. typhimurium, Escherichia coli* O157:H5 (ATCC), *C. rodentium* (ATCC), *B. fragilis*, or the single clone of S24-7 family of Bacteroidetes] were incubated with mouse mature α-defensin 5 (4.5 μM) in 200 μL PBS under aerobic or anaerobic conditions at 37°C for 1 h. And then the samples were lysed with equal volume AU-PAGE loading solution. The AU-PAGE was used to detect the integrity of mature α-defensin 5, and the gels were stained with Coomassie brilliant blue.

### Statistical Analysis

GraphPad Prism software was used for data analysis. For graphs, data are shown as mean ± SEM. Statistical significance was determined using a two-tailed unpaired Student *t*-test, *p ≤ 0.05, **p ≤ 0.01, ***p ≤ 0.001, ****p ≤ 0.0001, ns = nonsignificant.

## Results

### α-Defensins Promote *Bacteroides* Recovery After Antibiotic Intervention

**Figure 1:**
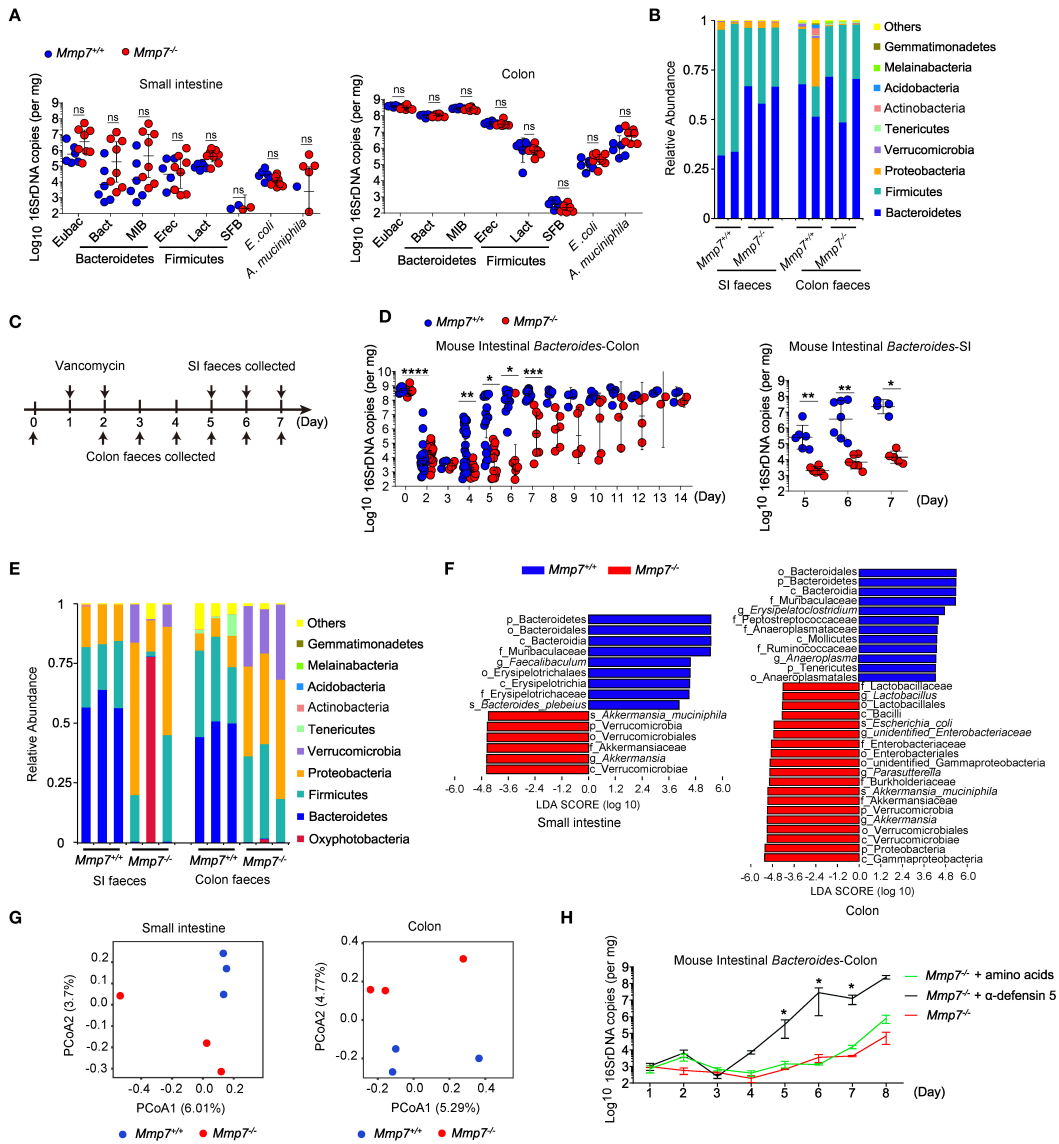
α-Defensins facilitate *Bacteroides* recovery after antibiotic challenges. **(A)** Quantitative PCR (qPCR) analysis of specific bacterial 16S rDNA in the small intestine and colon of *Mmp7*^+/+^ and *Mmp7*^−/−^ littermates under homeostasis conditions (*n* = 6–8). **(B)** 16S rDNA sequencing analysis of commensal diversity at the phylum level in the small intestine (SI) and colon of *Mmp7*^+/+^ and *Mmp7*^−/−^ littermates under homeostasis conditions (*n* = 2–3). **(C)** The experimental scheme for vancomycin administration. **(D)** qPCR analysis of the bacterial 16S rDNA of mouse intestinal *Bacteroides* in colon and SI of *Mmp7*^+/+^ and *Mmp7*^−/−^ littermates after vancomycin treatment (*n* = 5-6). **(E–G)** 16S rDNA sequencing analysis of commensal diversity in the SI and colon of *Mmp7*^+/+^ and *Mmp7*^−/−^ littermates at day 6 upon vancomycin treatment (*n* = 3). The relative abundances of bacterial taxa shown in **(E)**. LDA scores of the differentially abundant taxa shown in **(F)** (taxa with LDA score >4 and significance of α < 0.05 determined by Wilcoxon signed ranks test). Principal coordinate analysis of weighted Bray–Curtis shown in **(G)**. **(H)** *Mmp7*^−/−^ littermates given amino acids, mature α-defensin 5 and PBS for 1 week before vancomycin treatment. The mouse intestinal *Bacteroides* was analyzed by qPCR (*n* = 3–5). Data are pooled from multiple independent experiments **(A,D)**. Data are shown as mean ± SEM. Student *t*-test was performed; *p ≤ 0.05, **p ≤ 0.01, ***p ≤ 0.001, ****p ≤ 0.0001; ns, not significant (*p* > 0.05).

First, we investigated the effects of α-defensins on intestinal microbiota using MMP7-deficient mice that lacked mature α-defensins yet displayed normal intestinal architectures (Figures S1A, S1B) (14, 20). Microbiome analyses of multiple independent pairs of *Mmp7*^−/−^ and *Mmp7*^+/+^ littermates did not detect significant differences of *Bacteroides* or other bacterial groups in small intestine and colon between two genotypes (Figures 1A,B). The above results suggested a dispensable role for the MMP7-α-defensin axis in shaping commensal microbiota under the homeostatic conditions when mice were housed at the SPF animal facility of the authors' institution. To further investigate the plausible connections between α-defensins and microbiota, MMP7-deficient mice were subjected to various environmental challenges such as antibiotics treatments. As expected (27), in WT animals, treatments with vancomycin resulted in rapid depletion of *Bacteroides* population in gut lumen, from ~10^9^ copies to ~10^4^ copies, 2 days after treatment, and subsequently the *Bacteroides* population started to recover around day 4 (Figures 1C,D). In contrast, *Mmp7*^−/−^ mice displayed markedly compromised recovery of *Bacteroides* compared with the *Mmp7*^+/+^ littermates. When the *Bacteroides* loads in *Mmp7*^+/+^ mice almost reached the predepletion levels (~10^9^ copies in colon and ~10^7^ copies in small intestine) at day 7, the *Bacteroides* loads in *Mmp7*^−/−^ littermates remained several logs lower at 10^3^ to 10^7^ copies in colon and ~10^4^ copies in small intestine at day 7 and reached the predepletion levels around days 10 to 14 (Figure 1D). The defects in recovery were relatively specific to *Bacteroides* as several other bacterial groups examined did not show similar trends (Figures S2A–D). This phenotype was further confirmed by 16S rDNA sequencing of the fecal samples collected at day 6, which showed that while in *Mmp7*^+/+^ littermates, Bacteroidetes represented the most abundant phylum of bacterial community in small intestine and colon, Bacteroidetes was scarce in *Mmp7*^−/−^ mice (Figures 1E,F). In addition, the weighted UniFrac–principal coordinates analyses showed that the microbiome in *Mmp7*^−/−^ mice clustered separately from that in *Mmp7*^+/+^ littermates at day 6 (Figure 1G). To further demonstrate that facilitation of *Bacteroides* recovery by MMP7 was indeed due to its enzymatic activities on α-defensins, *Mmp7*^−/−^ mice were supplemented with synthetic mature α-defensin 5 or amino acid mixtures after vancomycin treatment. Exogenous α-defensins promoted *Bacteroides* recovery from vancomycin-mediated depletion and compensated for the loss of MMP7 (Figure 1H), implying that MMP7 and α-defensins were functionally coupled to exert these effects on *Bacteroides*. Taken together, these results revealed that α-defensins promoted intestinal *Bacteroides* recovery after antibiotics-induced dysbiosis.

### α-Defensins Enhance *Bacteroides* Colonization of Mucosal Niches in the Gut

**Figure 2:**
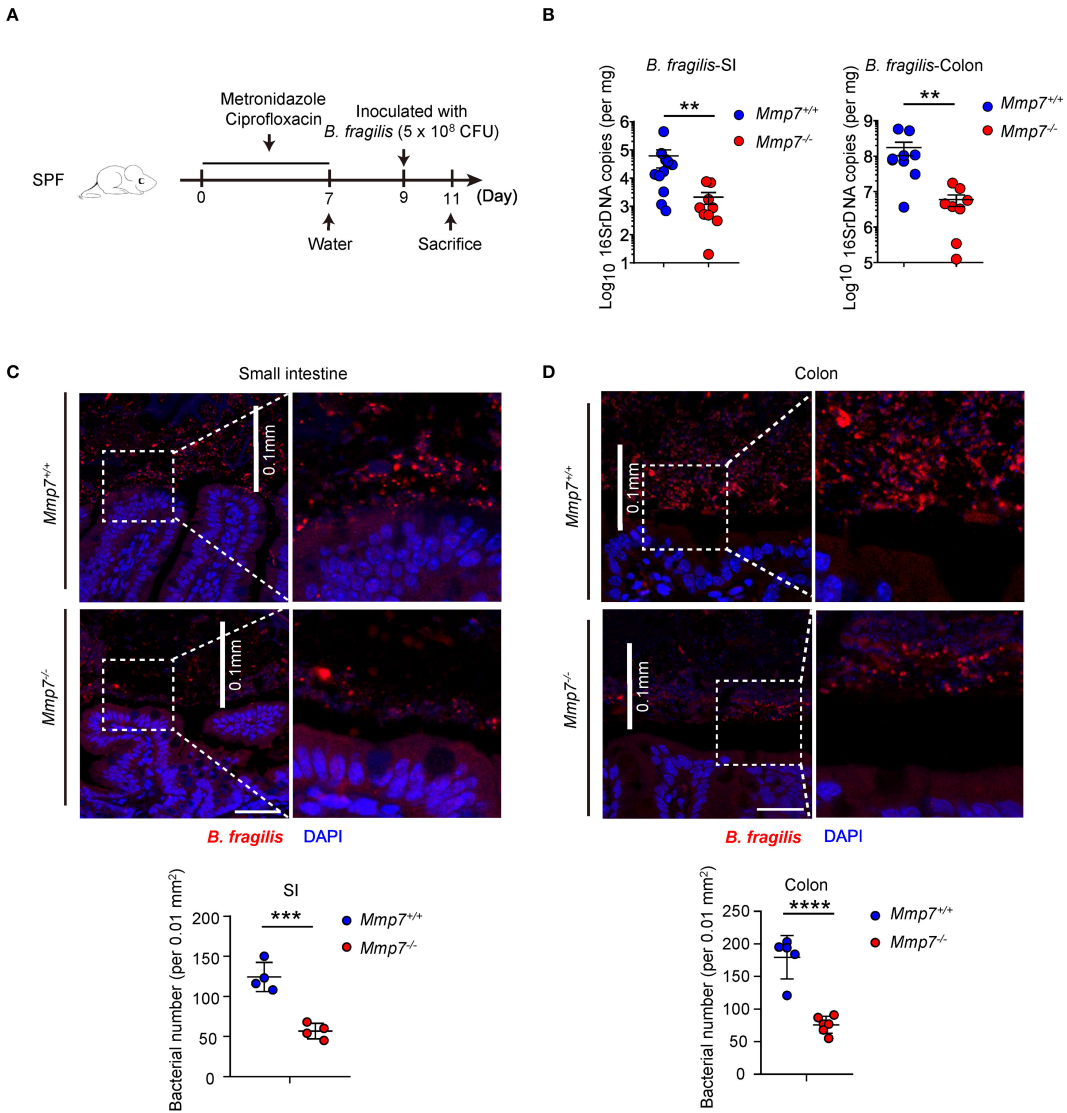
α-Defensins promote *B. fragilis* colonization of mucosal niches in the gut. **(A)** The experimental scheme for *B. fragilis* colonization in SPF mice. **(B)** *B. fragilis* colonization in *Mmp7*^+/+^ and *Mmp7*^−/−^ littermates were analyzed by qPCR (*n* = 8–10). **(C,D)** Fluorescent immunohistochemistry analysis of *B. fragilis* in the small intestine **(C)** and colon **(D)** from *Mmp7*^+/+^ and *Mmp7*^−/−^ littermates 2 days post gavage. Scale bar, 50 μm. The number of *B. fragilis* per 0.01 mm^2^ (0.1 × 0.1 mm; the white line indicates the linear distance of 0.1 mm) area from the surfaces of small intestine and colon (*n* = 4–5). Data are pooled from multiple independent experiments **(B)**. Data are shown as mean ± SEM. Student *t*-test was performed; **p ≤ 0.01, ***p ≤ 0.001, ****p ≤ 0.0001. Multiple tissues sections were from each mice, and each group contains more than 3 individual mice on average in **(C,D)**.

To investigate how α-defensins promote intestinal *Bacteroides* recovery after antibiotics treatment, we next tested whether α-defensins promote *Bacteroides* colonization to intestinal niches, and *Mmp7*^−/−^ and *Mmp7*^+/+^ littermates were pretreated with ciprofloxacin and metronidazole (7) and inoculated with *B. fragilis*, one of the most abundant species of the human *Bacteroides* genus (Figure 2A). Such experimental system allowed for tracking and visualization of the implanted bacterial species, which was not feasible for the murine endogenous *Bacteroides* populations. Two days after gavage, *Mmp7*^+/+^ mice showed higher colonization of *B. fragilis* than *Mmp7*^−/−^ littermates in small intestine and colon (Figure 2B), suggesting that α-defensins facilitated *B. fragilis* colonization in the gut. Building on previous studies that the intestinal microbiota occupies both mucosal and luminal niches during normal colonization (3), we next determined whether α-defensins regulated occupation of specific niches by *B. fragilis* during its colonization. Analyses of *B. fragilis* in ileal and colonic niches by confocal microscopy showed that the *B. fragilis* in *Mmp7*^+/+^ mice occupied more extensive epithelial surfaces than that in *Mmp7*^−/−^ littermates (Figures 2C,D). Collectively, these results showed that α-defensins promoted *B. fragilis* colonization of mucosal niches such as epithelial surfaces in the gut.

### α-Defensins Facilitate *Bacteroides* Adhesion to Epithelial Cells

**Figure 3:**
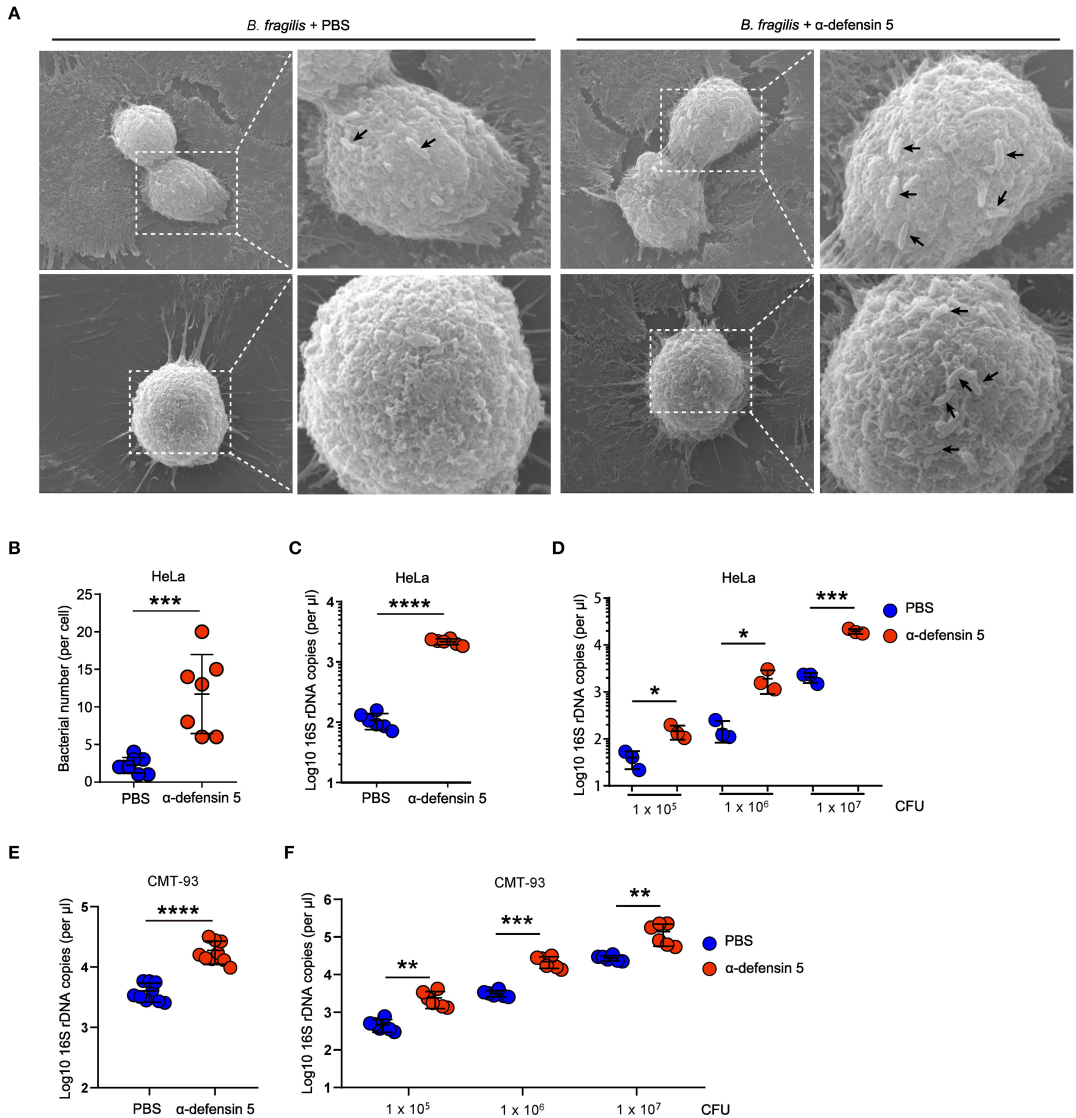
α-Defensin 5 facilitates adherence of *B. fragilis* to epithelial cells. **(A,B)** SEM analysis of *B. fragilis* (1 × 10^5^ CFUs) adhesion to HeLa cells with or without synthetic mouse mature α-defensin 5 (10 μM). The black arrow indicates the *B. fragilis* in the surface of HeLa cells. Quantifications are shown in **(B)** (*n* = 7). **(C)** qPCR analysis of *B. fragilis* (1 × 10^5^ CFUs) adhesion to HeLa cells with or without mouse mature α-defensin 5 (10 μM) (*n* = 6). **(D)** qPCR analysis of different numbers of *B. fragilis* adhesion to HeLa cells with or without mouse mature α-defensin 5 (*n* = 3). **(E)** qPCR analysis of *B. fragilis* (1 × 10^5^ CFUs) adhesion to CMT-93 cells with or without mouse mature α-defensin 5 (10 μM) (*n* = 9). **(F)** qPCR analysis of different numbers of *B. fragilis* adhesion to CMT-93 cells with or without mouse mature α-defensin 5 (*n* = 6). Data are pooled from two independent experiments **(A–C)**. Data are shown as mean ± SEM. Student *t*-test was performed; *p ≤ 0.05, **p ≤ 0.01, ***p ≤ 0.001, ****p ≤ 0.0001.

In order to investigate whether α-defensins directly promote *Bacteroides* colonization of epithelial niches by regulating bacterial adhesion, we next used HeLa cells to quantify bacterial adhesion as previously reported (11). The SEM results showed that synthetic mouse mature α-defensin 5–treated *B. fragilis* clustered on the surface of HeLa cells within 20 min of bacteria and HeLa coincubation, whereas control-treated *B. fragilis* minimally adhered to HeLa cells (Figures 3A,B). Consistent with these findings, quantification of the numbers of adherent *B. fragilis* demonstrated that α-defensin 5 enhanced *B. fragilis* adhesion to HeLa cells (Figure 3C). Likewise, mouse α-defensin 5 enhanced adhesion over a wide range of *B. fragilis* densities (Figure 3D). Enhanced adhesion by α-defensin 5 was further confirmed using CMT-93 cells, a mouse intestinal epithelial cell line (Figures 3E,F). Together, these results revealed that α-defensin 5 facilitated *B. fragilis* adhesion to epithelial cells, implying that α-defensins promote commensal *Bacteroides* colonization by enhancing bacterium-epithelium interaction.

### α-Defensins Are Non-microbicidal Against *Bacteroides*

**Figure 4:**
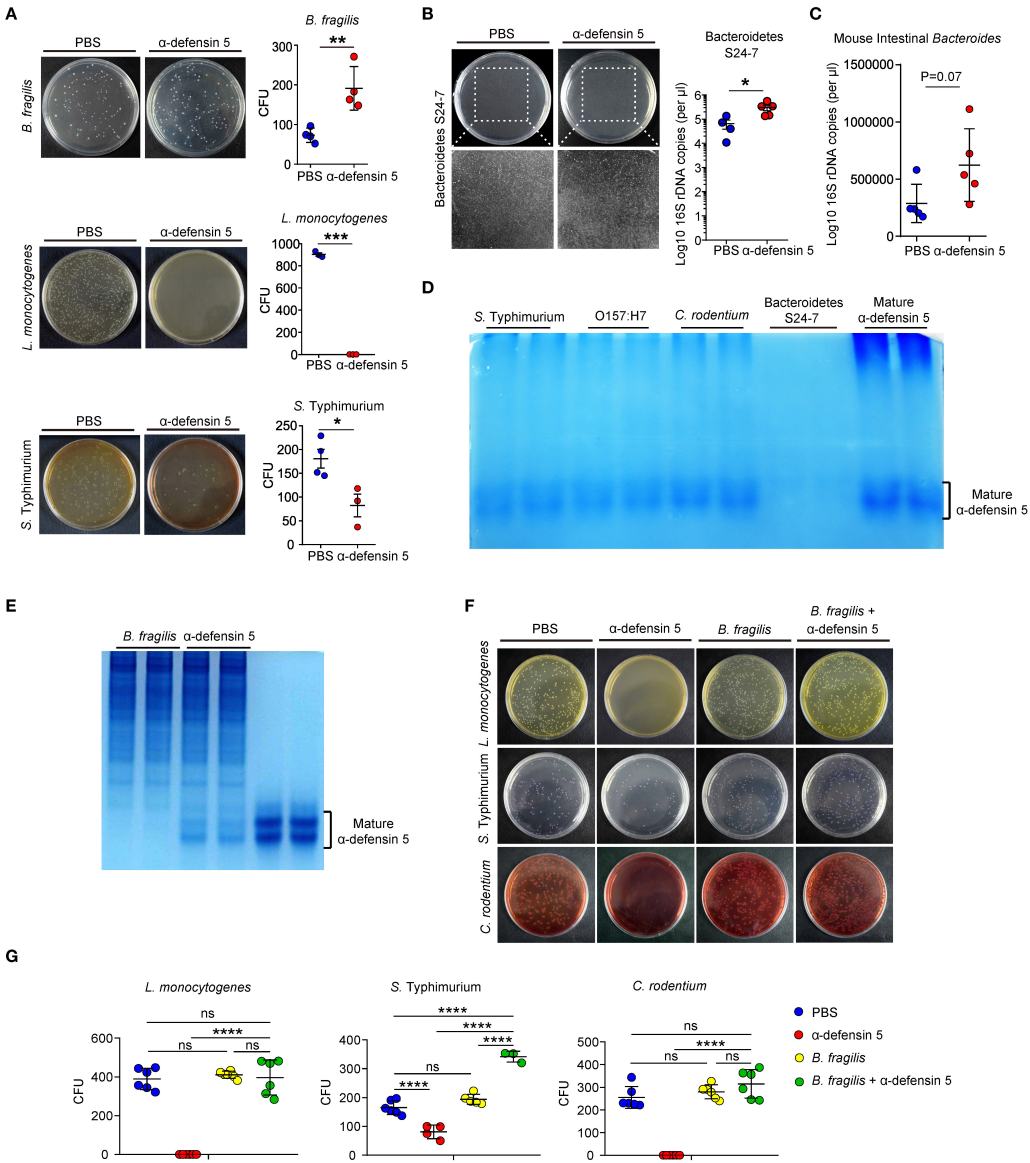
α-Defensin 5 does not exhibit antimicrobial activities against *Bacteroides*. **(A)** The antimicrobial activities of synthetic mature α-defensin 5 (4.5 μM) on *B. fragilis, L. monocytogenes*, and *S. typhimurium* were determined by CFUs (*n* = 3–4). **(B)** The antimicrobial activity of synthetic mature α-defensin 5 (4.5 μM) on a single clone of S24-7 family of Bacteroidetes was determined by qPCR analysis of specific bacterial 16S rDNA (*n* = 4–5). **(C)** The antimicrobial activity of synthetic mature α-defensin 5 (4.5 μM) on mouse intestine *Bacteroides* from feces-derived bacterial community was determined by qPCR analysis of specific bacterial 16S rDNA (*n* = 5). **(D)** 1 × 10^6^ CFUs *S. typhimurium, E. coli* O157:H7, *C. rodentium*, or a single clone of S24-7 family was incubated with mature α-defensin 5 (4.5 μM). The integrity of α-defensin 5 was detected by AU-PAGE. **(E)** 1 × 10^6^ CFUs *B. fragilis* was incubated with mature α-defensin 5 (4.5 μM). The integrity of α-defensin 5 was detected by AU-PAGE. **(F,G)** The antimicrobial activities of synthetic mature α-defensin 5 (4.5 μM) with or without preincubation of *B. fragilis* on *L. monocytogenes, S. typhimurium*, and *C. rodentium* were determined by CFUs. Data shown are representative for two to three independent experiments. Data are shown as mean ± SEM. Student *t*-test was performed; *p ≤ 0.05, **p ≤ 0.01, ***p ≤ 0.001, ****p ≤ 0.0001.

α-Defensins possess a wide range of microbicidal activities against Gram-positive and Gram-negative bacteria (28). However, data from the above *in vivo* experiments suggested that α-defensins were likely non-destructive for commensal *Bacteroides* (Figure 1). To directly assess the impact of α-defensins on *Bacteroides*, synthetic mature α-defensin 5 was coincubated with *B. fragilis, L. monocytogenes*, and *S. typhimurium in vitro*. Synthetic α-defensin 5 displayed potent bactericidal activities against pathogenic bacteria *L. monocytogenes* and *S. typhimurium* but not against *B. fragilis* on the agar plates (Figure 4A). To evaluate whether non-killing activities of α-defensins also apply to mouse endogenous Bacteroidetes, a single clone of S24-7 family from the Bacteroidetes phylum [also known as Muribaculaceae (29)] was isolated from mouse feces and maintained under anaerobic conditions (Figure S3). In line with the data obtained using *B. fragilis*, addition of α-defensin 5 did not reduce the growth of S24-7 organisms (Figure 4B) or diminish the abundance of intestinal *Bacteroides* when incubated with the collected stool samples (Figure 4C). To investigate the mechanisms underlying resistance of *Bacteroides* to α-defensin–mediated killing, we used AU-PAGE to detect the integrity of α-defensin 5 after coincubation with various bacterial species. Compared with pathogenic bacteria including *S. typhimurium, E. coli* O157:H7, and *C. rodentium*, incubation with a single clone of S24-7 family resulted in diminishment of α-defensin 5 (Figure 4D), implying potential inactivation and/or degradation of α-defensin 5. This effect of potential inactivation of α-defensin 5 was further confirmed by incubation with *B. fragilis*, which demonstrated partial inactivation of α-defensin 5 and reduced bactericidal activities against *L. monocytogenes, S. typhimurium*, and *C. rodentium* after preincubation with *B. fragilis* (Figures 4E–G). Thus, these findings suggested that commensal *Bacteroides* bacteria resisted α-defensin–mediated microbicidal action plausibly through reciprocal biochemical interactions between bacterial and defensin proteins.

## Discussion

α-Defensins are among the most evolutionarily ancient AMPs and highly expressed by Paneth cells in the small intestine (15). They help maintain gut homeostasis by forming a biochemical barrier that protects the host from infection and continuous exposure to potentially inflammatory stimuli (9). Here we presented unexpected *in vivo* and *in vitro* observations supporting a role for α-defensins in promoting *Bacteroides* recovery after antibiotic intervention by facilitating bacterial adhesion to the mucosal reservoirs such as epithelial surfaces (Figure S4).

A previous study using *Mmp7*^−/−^ and *DEFA5*-transgenic mice showed that α-defensins help maintain the proportions of Bacteroidetes yet diminish the proportions of Firmicutes at homeostasis (19). However, in our study, thorough analyses of bacterial communities associated with two intestinal locations in *Mmp7*^−/−^ and *Mmp7*^+/+^, littermates did not reveal prominent functions of α-defensins in shaping the compositions or diversity of Bacteroidetes or Firmicutes under homeostatic conditions. Although our data agreed with a minimal role of α-defensins in shaping homeostatic microbiota, we could not rule out the possibility that regulation of steady-state microbiota by α-defensins is influenced by a number of factors including geographic locations of mouse facilities, diet, and the immune status of mice. One study documented that mice housed in different rooms within the same animal facility harbored different gut microbiota and exhibited different barrier structures (30). Diet and immune status have been reported to influence biogeography of bacteria in the gut (3). Moreover, this speculation is supported by a report showing that shaping of microbiota composition by host genetic effects depends on community structure (31).

Specific niches such as crypts, mucus, and epithelial surfaces protect commensal species and serve as reservoirs to repopulate the lumen after environmental challenges (3, 5, 7), supporting the possibility that α-defensins may facilitate *Bacteroides* recovery by promoting bacterial colonization of mucosal niches in the gut. Interestingly, α-defensins promoted *Bacteroides* recovery after antibiotic treatment but did not regulate the population of *Bacteroides* at homeostasis, which could be plausibly explained by the presence of mucosal reservoirs for *Bacteroides*. Under normal conditions, *Bacteroides* extensively occupy the gut lumen (3) because of the fact that rapidly proliferating bacteria from luminal reservoirs continuously repopulate the lumen utilizing energy sources from diet-derived nutrients (32). On such occasion, mucosal reservoirs of *Bacteroides* may not be required for repopulating the lumen. Nevertheless, when majority of the luminal *Bacteroides* bacteria are depleted by antibiotics, the consequences of regulation of mucosal reservoirs by α-defensins could possibly be manifested, as the bacterial cells preserved in mucosal reservoirs may represent the predominant source for repopulating the lumen. In line with a previous report (33), our study implies that epithelia surfaces act as a dominant *Bacteroides* reservoir, which is facilitated not only by IgA but also by α-defensins.

α-Defensins are the most abundant and diverse AMP families in the small intestine (12, 15), but it remains a mystery why the mucosal immune system is evolved to invest considerable amounts of energy to produce such high quantities of α-defensins on a daily basis, whereas mice deficient in mature α-defensins are viable and do not display any gross physical or behavioral abnormalities under homeostatic conditions (20, 34). Our previous study has suggested an important role for α-defensins in protecting host from pathogenic bacterial infection under nutrient-deprived conditions, indicating that environmental stresses may reveal the otherwise masked functions of α-defensins (20). In line with these findings, the current study demonstrates that instead of affecting the commensal bacterial community at homeostasis, α-defensins promote *Bacteroides* recovery upon environmental stresses such as antibiotic challenges. In junction with the previously reported role of human α-defensin 5 in promoting *Shigella* infection (11), we propose that α-defensins possibly play an evolutionarily conserved role in broadly impacting adhesion of commensal or pathogenic bacteria to intestinal epithelia cells to shape microbiota ecology and thus influencing the microbiota-associated diseases such as IBDs.

## Data Availability Statement

The raw data supporting the conclusions of this article will be made available by the authors, without undue reservation.

## Ethics Statement

The animal study was reviewed and approved by Institutional Animal Care and Use Committee (IACUC) at Tsinghua University.

## Author Contributions

JO designed research, performed experiments, analyzed data, and wrote the manuscript. SL provided critical reagents for some experiments. X-KG and XH conceptualized the project, designed research, supervised experiments, and wrote the manuscript. All authors contributed to the article and approved the submitted version.

## Conflict of Interest

The authors declare that the research was conducted in the absence of any commercial or financial relationships that could be construed as a potential conflict of interest.
