# Supplementary material for: α-Defensins Promote Bacteroides Colonization on Mucosal Reservoir to Prevent Antibiotic-Induced Dysbiosis
Source: Front Immunol. 2020 Sep 9;11:2065. doi: 10.3389/fimmu.2020.02065 (PMC7509133; doi:10.3389/fimmu.2020.02065)
Supplement: Supplementary file 1 [file Table_1.DOCX]

**
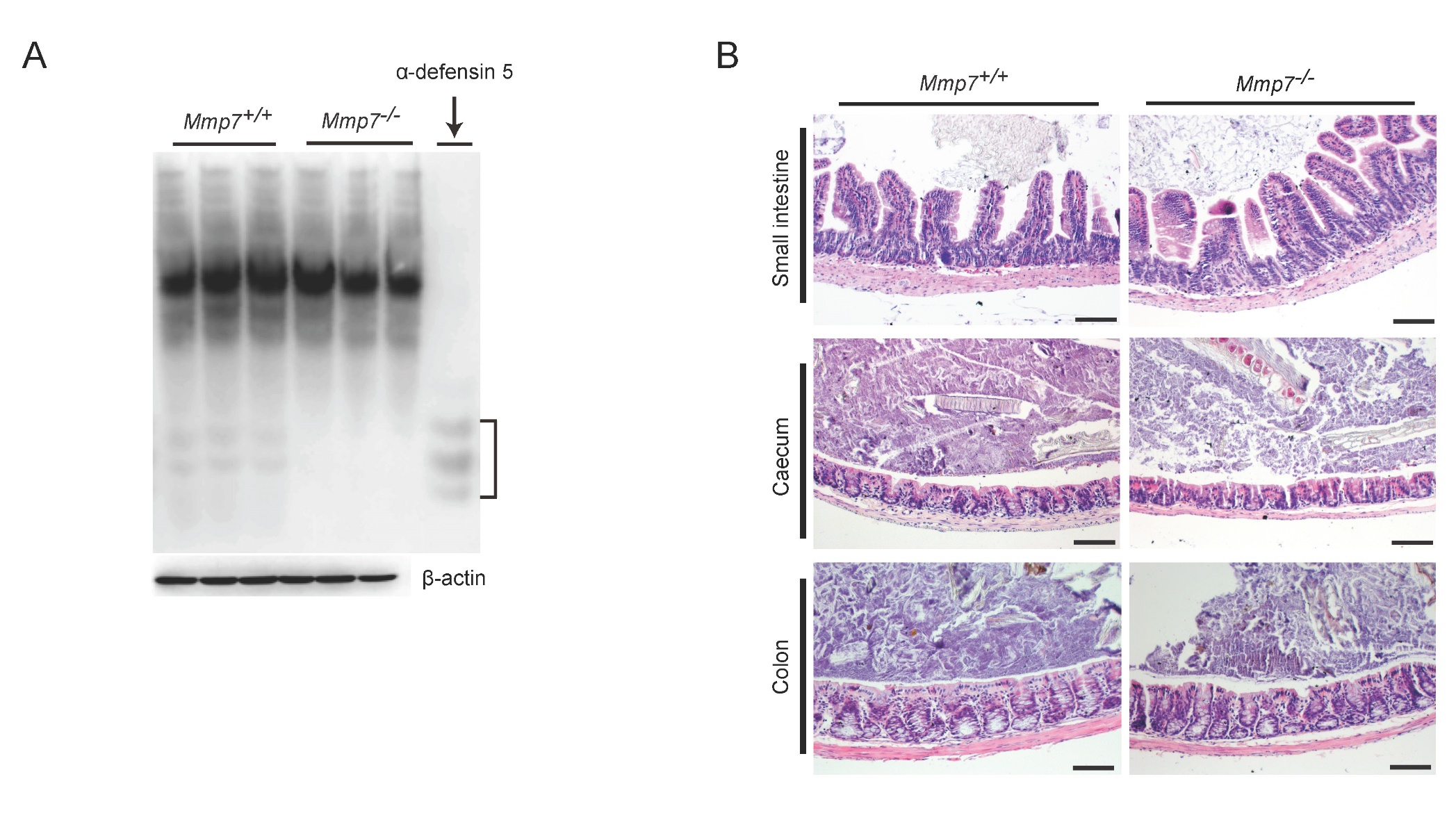
**

**Figure S1. Analysis of mature α-defensins and the histology of *Mmp7^+/+^* and *Mmp7*^–/–^ at homeostasis**

1. Immunoblotting analysis of mature α-defensins resolved by AU-PAGE in the ileum of *Mmp7^+/+^* and *Mmp7*^–/–^ littermates. Synthetic mature α-defensin 5 served as a positive control.
2. H&E staining of small intestine, caecum and colon sections from *Mmp7^+/+^* and *Mmp7*^–/–^ littermates.

**
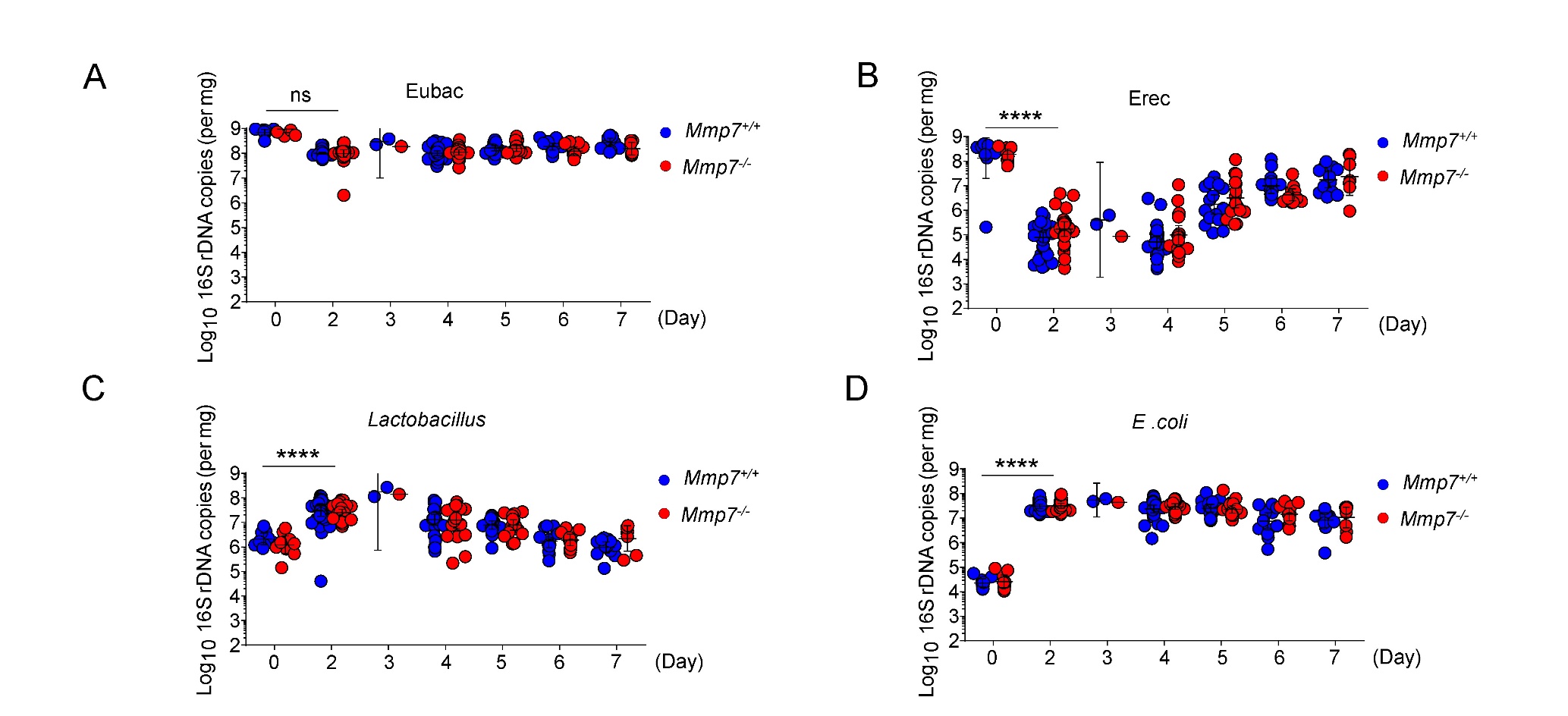
**

**Figure S2. Analysis of other representative commensal microbiota in *Mmp7^+/+^* and *Mmp7*^–/–^ littermates post vancomycin treatment**

qPCR analysis of representative commensal microbiota (A-D) in the colon from *Mmp7^+/+^* and *Mmp7*^–/–^ littermates in recovery process post vancomycin treatment (n = 5-8 per group).

**
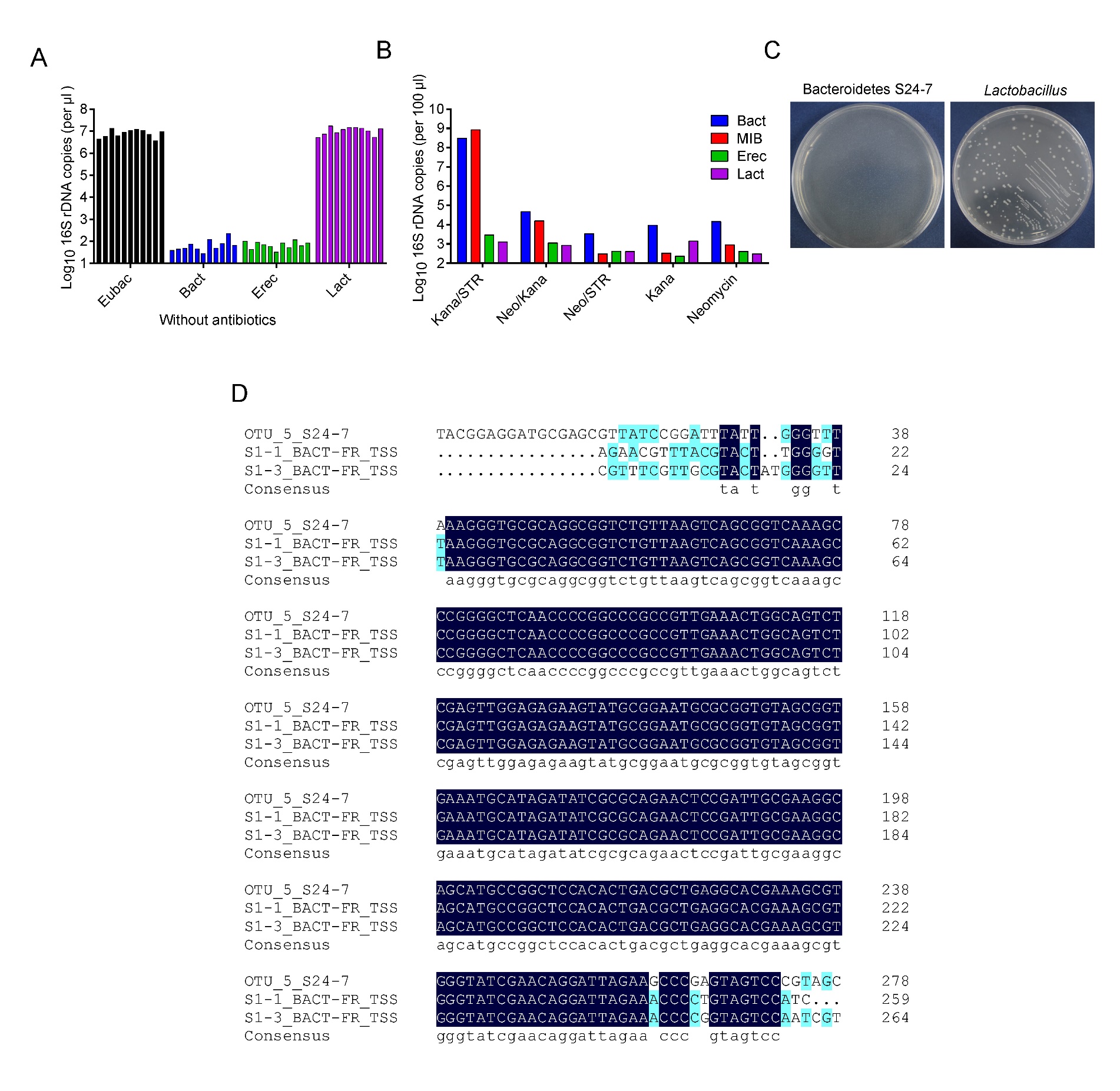
**

**Figure S3. Isolation and identification of the mouse Bacteroidetes S24-7 bacteria**

(A) qPCR analysis of isolated bacteria from faeces growing on the Wilkins Chalgren anaerobic agar.

(B) qPCR analysis of isolated bacteria from faeces growing on the Wilkins Chalgren anaerobic agars containing kanamycin/streptomycin, neomycin/kanamycin, neomycin/streptomycin, kanamycin, or neomycin, respectively (n=8-11 per group).

(C) Bacterium forming the colony from a single clone of family S24-7 of Bacteroidetes (left) and a single clone of *Lactobacillus* (right).

(D) Identification of a potential single clone of family S24-7 of Bacteroidetes by sequence alignment.

**
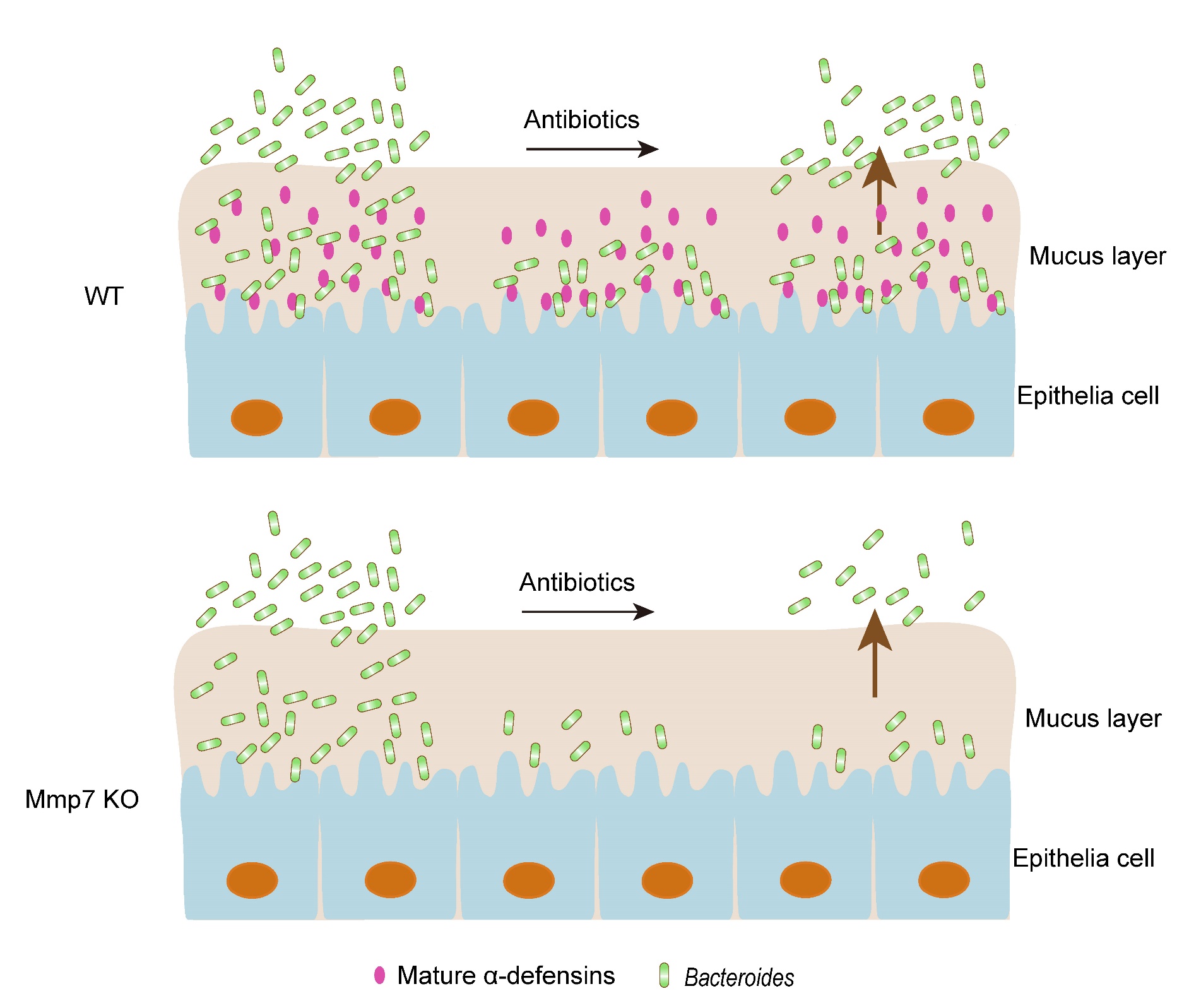
**

**Figure S4. α-defensins promote commensal *Bacteroides* colonization during the recovery from antibiotic-mediated depletion**

A working model for promotion of *Bacteroides* colonization by α-defensins after antibiotic challenge. Under normal conditions, *Bacteroides* highly occupy the intestinal lumen when the rapidly proliferated bacteria from luminal reservoirs can continuously repopulate the lumen. Loss of mature α-defensins play minimal roles in repopulating the lumen community in Mmp7-KO (knock-out) mice. However, when most of the *Bacteroides* bacterium in the lumen were depleted by antibiotic treatment, the impacts of mucosal reservoirs regulated by α-defensins on the gut *Bacteroides* population could be dominant, as the mucosal reservoirs preserved bacterial cells may represent the predominant source for repopulating the lumen. On this condition, α-defensins can promote *Bacteroides* recovery after antibiotic intervention by facilitating bacterial adhesion to the mucosal reservoirs such as epithelial surfaces.

**Supplementary Table 1:**

**Primers used in this study**

**Bacterial 16S rRNA gene primers:**

| **Target** | **Forward Primer** | **Reverse Primer** |
| --- | --- | --- |
| Eubacteria (Universal) | ACTCCTACGGGAGGCAGCAGT | ATTACCGCGGCTGCTGGC |
| *Bacteroides* (Bact) | GGTTCTGAGAGGAGGTCCC | CTGCCTCCCGTAGGAGT |
| Mouse Intestinal  *Bacteroides* (MIB) | CCAGCAGCCGCGGTAATA | CGCATTCCGCATACTTCTC |
| Lactobacillus/Enterococcus  Group (Lact) | AGCAGTAGGGAATCTTCC | CACCGCTACACATGGA |
| *Eubacterium rectale*/  *Clostridium coccoides*  group (Erec) | ACTCCTACGGGAGGCAGC | GCTTCTTAGTCAGGTACCGTCA |
| Segmented filamentous  bacteria (SFB) | GACGCTGAGGCATGAGAGCA | GACGGCACGGATTGTTATTC |
| *E. coli* | CATGCCGCGTGTATGAAGAA | CGGGTAACGTCAATGAGCAAA |
| *A. muciniphila* | CAGCACGTGAAGGTGGGGAC | CCTTGCGGTTGGCTTCAGAT |
| *B. fragilis* | TCRGGAAGAAAGCTTGCT | CATCCTTTACCGGAATCCT |
